# *Musca domestica* Cecropin (Mdc) Alleviates *Salmonella typhimurium*-Induced Colonic Mucosal Barrier Impairment: Associating With Inflammatory and Oxidative Stress Response, Tight Junction as Well as Intestinal Flora

**DOI:** 10.3389/fmicb.2019.00522

**Published:** 2019-03-15

**Authors:** Lun Zhang, Shuiqing Gui, Zhaobo Liang, Along Liu, Zhaoxia Chen, Yanan Tang, Mingzhu Xiao, Fujiang Chu, Wenbin Liu, Xiaobao Jin, Jiayong Zhu, Xuemei Lu

**Affiliations:** ^1^Guangdong Provincial Key Laboratory of Pharmaceutical Bioactive Substances, Guangdong Pharmaceutical University, Guangzhou, China; ^2^Intensive Care Unit, Shenzhen Second People’s Hospital, The First Affiliated Hospital of Shenzhen University, Shenzhen, China

**Keywords:** *Musca domestica* cecropin, *Salmonella typhimurium*, colonic mucosal barrier, inflammation, oxidative stress, tight junctions, intestinal flora

## Abstract

*Salmonella typhimurium*, a Gram-negative food-borne pathogen, induces impairment in intestinal mucosal barrier function frequently. The injury is related to many factors such as inflammation, oxidative stress, tight junctions and flora changes in the host intestine. *Musca domestica* cecropin (Mdc), a novel antimicrobial peptide containing 40 amino acids, has potential antibacterial, anti-inflammatory, and immunological functions. It remains unclear exactly whether and how Mdc reduces colonic mucosal barrier damage caused by *S. typhimurium*. Twenty four 6-week-old male mice were divided into four groups: normal group, control group (*S. typhimurium*-challenged), Mdc group, and ceftriaxone sodium group (Cs group). HE staining and transmission electron microscopy (TEM) were performed to observe the morphology of the colon tissues. Bacterial load of *S. typhimurium* in colon, liver and spleen were determined by bacterial plate counting. Inflammatory factors were detected by enzyme linked immunosorbent assay (ELISA). Oxidative stress levels in the colon tissues were also analyzed. Immunofluorescence analysis, RT-PCR, and Western blot were carried out to examine the levels of tight junction and inflammatory proteins. The intestinal microbiota composition was assessed via 16s rDNA sequencing. We successfully built and evaluated an *S. typhimurium*-infection model in mice. Morphology and microcosmic change of the colon tissues confirmed the protective qualities of Mdc. Mdc could inhibit colonic inflammation and oxidative stress. Tight junctions were improved significantly after Mdc administration. Interestingly, Mdc ameliorated intestinal flora imbalance, which may be related to the improvement of tight junction. Our results shed a new light on protective effects and mechanism of the antimicrobial peptide Mdc on colonic mucosal barrier damage caused by *S. typhimurium* infection. Mdc is expected to be an important candidate for *S. typhimurium* infection treatment.

## Introduction

*Salmonella typhimurium* is an important Gram-negative food-borne pathogen with a broad host range including humans and animals, and causes diseases ranging from gastroenteritis and diarrhea to life-threatening systemic infections (Wan et al., 2018), resulting in one million human deaths annually (Balasundaram et al., 2017). Owing to their metabolic versatility, *Salmonella* can colonize as multicellular aggregates on various surfaces, enhancing virulence levels through biofilm formations that often resist antibiotic therapy (Faccone et al., 2018; Kuang et al., 2018; Ridge et al., 2018; Wan et al., 2018).

*Salmonella typhimurium* challenge induces impairment in intestinal mucosal barrier function (Zhang et al., 2012; Boyer et al., 2015). The epithelial barrier is one of the most important components of the intestinal mucosal barrier against macromolecular transmission (Collado-Romero et al., 2012). The epithelial cells form a continuous intact physical epithelial barrier with interspersing tight junctions (TJs) between each cell. Disruption of TJs results in increased permeability to luminal antigens and bacteria, degrading mucosal barrier function. TJs can be altered by several bacterial pathogens through modification of TJ proteins such as occludin, claudin-1, and ZO-1. Infection of polarized epithelial cell monolayers by *S. typhimurium* may disrupt TJ structure and function, and subsequently disrupts epithelial barrier integrity (Zhang et al., 2012). Inflammation and oxidative stress are considered as the essential mechanism underlying the pathophysiology of intestinal injury (Di Sabatino et al., 2016). A relative increase in some inflammatory factors may attenuate zonula occluding protein expression, leading to intestinal barrier dysfunction, and promotion of intestinal inflammation in the colonic mucosa (Ogawa et al., 2018).

Intestinal microflora play a crucial role in host defense as demonstrated by their ability to modulate both innate and acquired immunity at the local as well as systemic levels (Colliou et al., 2017). Intestinal flora may also contribute to the development and progression of cancer (Loo et al., 2017). Most importantly, changes in microbe populations may be closely related to the degree of intestinal inflammation (Frank et al., 2011), which is one of the characteristics of *S. typhimurium* infection (Bronner et al., 2018). Host microbiota likely play a role in resistance to, and may also be indirectly responsible for some of the consequences of *S. typhimurium* infection (Argüello et al., 2018). Resistance to *S. typhimurium* by adjusting intestinal flora was reported long ago or recently (Baba et al., 1991; Deriu et al., 2013). Oral administration of probiotics has been demonstrated to increase paneth cells and intestinal antimicrobial activity, which is the main intestinal cell responsible for the immunoreactive antimicrobial peptide (AMP) production. This peptide helps to stabilize the intestinal barrier, while promoting the stability of intestinal microbial flora (Cazorla et al., 2018). It was also reported that the enhanced activity of AMPs by statins in *Salmonella*-infected IECs could protect the host against infection, and modulation of pro-inflammatory responses could prevent the detrimental effects of severe inflammation in the host (Huang and Huang, 2018). AMPs serve to protect the intestinal barrier, but the mechanism of AMP activity remains unclear, especially for regulating intestinal flora.

Housefly (*Musca domestica*) larvae have been used clinically to treat ecthyma, decubital necrosis, lip boil, and osteomyelitis since 1368 Anno Domini (Ming/Qing Danysty) in China (Hou et al., 2007). *Musca domestica* cecropin (Mdc), a novel AMP containing 40 amino acids, has been cloned and characterized from housefly larvae, and was found to have a significant potency against *S. typhimurium* in our previous studies (Lu et al., 2010). However, it is unclear whether Mdc has potent protection against the intestinal barrier impairment in mice colonic mucosal. Moreover, the precise protective mechanism(s) of Mdc remain unknown. Thus, the present study aimed to investigate the effect and mechanisms of Mdc on colonic mucosal barrier damage caused by *S. typhimurium* infection.

## Materials and Methods

### Peptide and Material

*Musca domestica* cecropin (Mdc) (Purity 97.15%; molecular weight: 4301.59 Da) was chemically synthesized by conventional Fmoc solid-phase synthetic method (Beijing Scilight Biotechnology Ltd., Beijing, China) (Lu et al., 2015). Streptomycin sulfate was purchased from Amresco Inc, United States. Ceftriaxone sodium (1 g/bottle) was obtained from Guangdong succhi pharmaceutical (group) Ltd. All other chemicals were standard commercial products with analytical reagent grade.

### Microorganisms and Medium

*Salmonella typhimurium* GIM1.237 was obtained from Guangdong culture collection center. The compositions of medium from Guangdong Huankai Microbial Sci. & Tech. Co., Ltd in this work are as follows: Luria-Bertani (LB) medium [0.5% yeast extract (w/v), 1% tryptone, and 1% NaCl] and tryptone soybean broth (TSB) medium [1.7% Pancreatic digest of casein (w/v), 0.3% Enzymatic digest of soya bean, and 0.5% Sodium chloride, 0.25% Dipotassium hydrogen phosphate, 0.25% Glucose] were employed for pre-incubation of the test bacteria. The bacteria were grown overnight to an optical density at 600 nm (OD600) of 0.8–1.0 prior to appropriate dilution. The streptomycin resistant *S. typhimurium* suspension with a concentration of 0.7–0.75 × 10^9^ CFU/mL was prepared and the mice were given gavage with a dose of 300 *μ*L each.

### Animals and Experimental Design

**FIGURE 1:**
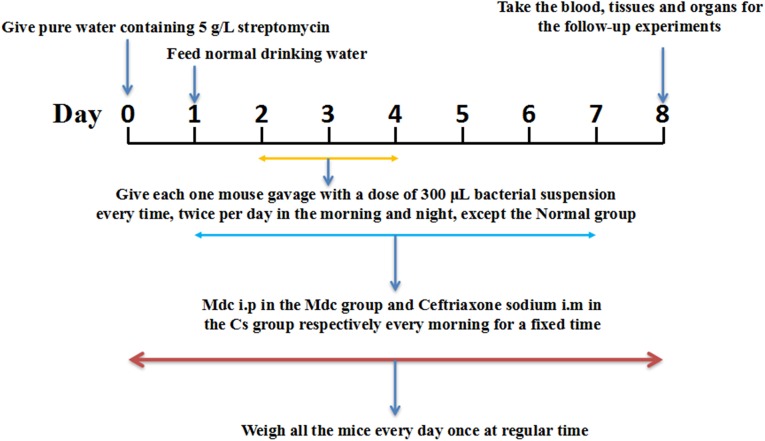
The animal grouping design.

C57BL6 mice, male, were obtained from Center for Experimental Animals, Guangdong Province [Guangzhou, China approval number SCXK (Yue) 2013-0002]. Twenty four 6-week-old male mice were housed (eating and drinking *ad libitum*) under controlled conditions (room temperature 23 ± 2°C; relative humidity 55 ± 5%; 12-h dark/12-h light cycle) for 1 week. The use of the animals and experimental protocols were approved by the Guidelines for the Care and Use of Experimental Animals, the Guangdong Pharmaceutical University [SYXK (Yue) 2012-0125] and the Guangdong Pharmaceutical University Animal Care and Use Committee, China. All the mice were randomly divided into four groups of six each: the normal group, the control group, the Mdc group, and the Cs group. Animal grouping design is shown in Figure 1. One day before the start of the trial, the mice in each group were given water containing 5 g/L streptomycin, and then returned to normal drinking water after 24 h. During the seven-day experiment, the mice were given medicine (Mdc i.p in the Mdc group and Cefatriaxone sodium i.m in the Cs group, respectively) every morning for a fixed time. From the second to the fourth day, except the normal group, the rest of the groups were given gavage with a dose of 300 *μ*L streptomycin resistant *S. typhimurium* suspension twice a day, morning and night. Blood and a variety of tissues were removed for the follow-up experiments. All animals were weighed and recorded at fixed times every day during the whole animal feeding period. Image-Pro Plus 6.0 was used for the analysis of intestinal thickness.

### Bacterial Load of *S. typhimurium* in Colon and Analysis of Bacterial Translocation in Liver and Spleen

A stool of 20 mg from each mouse’s colon was taken and homogenized in 1 mL sterile PBS solution. Then the homogenate was diluted and homogenate diluent of 100 *μ*L was coated on the LB agar plate containing 50 *μ*g/mL streptomycin. After 24 h incubation at 37°C, the total number of colonies on the plate and the content of *S. typhimurium* in every gram of feces were calculated. To investigate bacterial translocation after *S. typhimurium* infection, the bactrial load in the liver and spleen was determined separately. Liver and spleen tissues were washed with normal saline immediately after removal from mice and stuck dry with filter paper. A tissue of 100 mg from the same site was placed in a centrifugal tube containing pre-cooled 1 mL PBS solution and homogenized. Homogenate of 100 *μ*L was coated on the normal LB agar plate. After incubation at 37°C for 24 h, the actual bacterial content in each gram of tissue was counted.

### Analysis of Colonic Morphology

Intestinal tissues of the middle colon from six mice in each group were examined. To evaluate the histological changes in the intestines, full thickness sections of the middle colon were excised, irrigated gently to dislodge intestinal contents with normal saline, immediately fixed in 4% paraformaldehyde solution, and embedded in paraffin. Samples were crosscut and stained with hematoxylin-eosin (H&E). Photomicrographs of sections were observed with a light microscope and taken with a digital camera (DFC495 Digital camera Leica, German), scored in a blinded fashion by three investigators. Histological damage was scored using the criteria of Appleyard and Wallace (Appleyard and Wallace, 1995), including loss of mucosal architecture [0, 1, 2, or 3 (absent, mild and severe)], cellular infiltration [0, 1, 2, or 3 (absent, mild and severe)], muscle thickening [0, 1, 2, or 3 (absent, mild and severe)], crypt abscess formation (0 or 1 (absent or present)], and goblet cell depletion [0 or 1 (absent or present)]. The sum of scores in each area is the final score and the final scores of the three investigators were averaged. Based on the average scores, the statistics data for histological damage were obtained.

### Transmission Electron Microscopy (TEM)

To investigate the shape changes of intestinal mucosa after treatment with Mdc, we observed the ultrastructure of the colonic epithelium obtained by TEM. Distal colonic tissues were sliced into 1-mm^3^ cubes and immediately fixed in 0.1 M phosphate buffer containing 2.5% glutaraldehyde at 4°C for 12 h. After washing samples with 0.1 M phosphate buffer three times for 30 min, we fixed them in 1% osmium tetroxide for 2 h, and subsequently treated with 2% uranyl acetate for 1 h before dehydration in an ethanol series and embedded in epoxy resin. Finally, the embedded samples was sectioned and examined in a JEM1400 (Jeol, Japan) TEM. Microphotographs were taken with a digital camera Bioscan 792 (Gatan Inc., United States) and scored in a blinded fashion by three investigators. For the convenience of presentation, according to damage degree of microvilli, tight junction and epithelial cells, the mucosal changes in TEM are graded as follwing: (1) The score of complete and homogeneous microvilli is 0; the scores of short, sparse and curled microvilli in varying degrees are 1 (slight), 2 (moderate) and 3 (severe), respectively; the scores of microvilli falling off are 4 (mild) and 5 (severe), respectively. (2) The score of complete tight connection is 0 (close interconnection between cells); the scores of damaged tight junction are 1 (mild) and 2 (severe), respectively; the score of loss of tight junction is 3. (3) The score of normal epithelial cells is 0; the scores of structural damage of epithelial cells are 1 (slight), 2 (moderate) and 3 (severe), respectively. The sum of scores in each area is the final score and the final scores of the three investigators were averaged. Based on the average scores, the statistics data for histological damage were obtained.

### Enzyme Linked Immunosorbent Assay (ELISA)

To determine the concentration of TNF-α, IFN-γ, IL-6, and IL-10 in serum of all groups, ELISA Kits (Beijing 4A Biotech Co., Ltd) was used. The kit assays were carried out according to the manufacturer’s instructions.

### Detection of MPO, NAG, MDA, and GSH-Px in Colon

Colonic MPO, NAG, MDA, and GSH-Px activities were determined by using MPO Detection Kit, NAG Detection Kit, MDA Detection Kit, and GSH-Px Detection Kit, respectively. All the Kits were acquired from Nanjing Jiancheng Bioengineering Institute. The colon tissues were homogenized using 0.86% of cold saline as homogenizing medium before testing the samples complying with the instructions.

### Immunofluorescence Analysis

The distal colon samples were fixed in 4% paraformaldehyde solution, embedded in paraffin and cut into slices transversely, the steps before dewaxing were the same as those of hematoxylin-eosin (H&E) dyeing. For immunofluorescence analysis of Claudin-1 (Abcam UK), Occludin (Abcam UK), and ZO-1 (Abcam UK), COX-2 (Abcam UK), INOS (Abcam UK), P65 (Abcam UK), antigen retrieval was performed by heating the slices for 20 min in boiling water and non-specific binding sites were blocked with PBS containing 1% w/v BSA for 1 h at 37°C. The primary antibodies (1:200) above were added to the corresponding samples to incubate overnight at 4°C. After washing with PBS for three times, sections were incubated with the secondary antibody, Alexa Fluor 488-conjugated or Cy3-conjugated goat anti-rabbit IgG (1:200; Beyotime, China) at room temperature for 1 h in darkness. Eventually, DAPI was used to stain the nucleus. Images were taken under a fluorescence microscope (Leica, German) immediately. Fluorescence intensity was analyzed by using Image J.

### Real-Time Quantitative PCR Detection

RNA samples were extracted from the distal colon. Total RNA isolation and cDNA synthesis by reverse transcription were conducted using Trizol reagent (Invitrogen Corporation, United States) and PrimeScript^TM^ RT reagent Kit with gDNA Eraser (Takara Biotechnology Co. Ltd., Japan), respectively. The mRNA levels of individual genes were measured by real-time PCR using the SYBR Premix Ex Taq Kit (Takara Biotechnology Co. Ltd., Japan) in the CFX Connect fluorescence quantitative PCR detection system (BIO-RAD, United States). The 25 *μ*L PCR reaction mixture per reaction consists of 12.5 *μ*L SYBR Premix Ex Taq II (Tli RNaseH Plus) (2×), 1 *μ*L Forward Primer (10 *μ*M), 1 *μ*L Reverse Primer (10 *μ*M), 2 *μ*L reaction solution (cDNA), and 8.5 *μ*L Sterilized purified water. Shuttle PCR standard protocol is as following: Stage 1 is the initial denaturation of one cycle at 95°C for 30 s; Stage 2 is the PCR reaction of 40 cycles at 95°C for 5 s and 60°C for 30 s; Stage 3 is the Dissociation. Data were analyzed according to the comparative threshold cycle (Cq) method and normalized to an endogenous reference, β-actin. The primers used in this experiment were listed in Table 1. Relative expression levels of inflammation related (INOS, COX-2, P65) and barrier function related genes (ZO-1, Claudin-1, Occludin) were analyzed in colon tissues.

**Table 1:** The primers used in this experiment.

Gene name	Primer sequence (from 5′ end to 3′ end)	Product size (bp)
ZO1-F	CCAGCAACTTTCAGACCACC	154
ZO1-R	TTGTGTACGGCTTTGGTGTG	
Occludin-F	GCTTACAGGCAGAACTAGACG	142
Occludin-R	TCTGCAGATCCCTTAACTTGC	
Claudin1-F	TCGACTCCTTGCTGAATCTGA	153
Claudin1-R	TCCACATCTTCTGCACCTCA	
P65-F	TCTTCTTGCTGTGCGACAAG	177
P65-R	GCATGGAGACTCGAACAGGA	
INOS-F	ACAGGAACCTACCAGCTCAC	201
INOS-R	CGACCTGATGTTGCCATTGT	
COX2-F	AGGTCATTGGTGGAGAGGTG	192
COX2-R	CCTGCTTGAGTATGTCGCAC	
β-actin-F	AGAGGGAAATCGTGCGTGAC	138
β-actin-R	CAATAGTGATGACCTGGCCGT	


### Western Blot Analysis

Distal colons were ground and lysed with RIPA lysis buffer (Beyotime, China) with 1 mM PMSF (Beyotime, China). The protein content of each tissue was quantified and diluted and 20 *μ*g of protein was loaded in each lane of a 5/12% or a 5/8% SDS-PAGE gel. The protein was separated by SDS-PAGE and transferred to nitrocellulose membrane. The membrane was blocked with 5% (w/v) defatted milk in TBS and incubated with a primary antibody, P65 (1:1000), COX-2 (1:1000), INOS (1:1000), ZO-1 (1:1500), Claudin-1 (1;1000), Occludin (1:50000), and overnight at 4°C. The above-mentioned primary antibodies were from Abcam and the manufacturer’s instructions were referred to when determining the appropriate dilution for each antibody. HRP-conjugated secondary antibody (1:1000) (Beyotime, China) was applied before the proteins were visualized via chemiluminescence. Images were collected with Tanon-5200 (Shanghai Tanon Technology Co., Ltd, China), for quantification of protein levels, appropriate film exposures were scanned and the density of bands was determined with Image J and normalized to band intensity for GAPDH.

### Intestinal Flora Analysis

On the eighth day of the experiment, the three fecal samples from six mice in each group were collected and marked for sequencing, respectively, and the sequencing work was completed by the Novogene Corporation (Beijing, China).

### Statistical Analysis

Data were analyzed with the use of a 2-tailed unpaired Student’s *t* test. *P* ≤ 0.05 was considered to be significant. Each mouse was considered as an experimental unit. Data are expressed as means ± SEMs. Statistical analysis was performed using GraphPad Prism version 7.00.

## Results

### Model Building Evaluation

**FIGURE 2:**
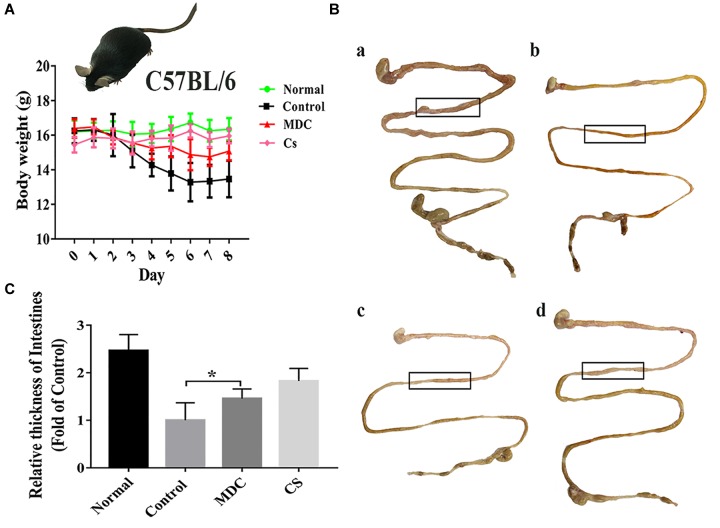
Mdc could significantly improve weight loss and intestinal thinning after infection. Changes in body weight of mice during modeling period **(A)**, comparison of intestinal appearance among normal group, control group, Mdc group, and Cs group **(B)**, and statistical analysis for thickness of intestines **(C)**. Values are means ± SEMs, *n* = 6, ^∗^*p* ≤ 0.05.

The infection model was established successfully by intragastric administration of streptomycin resistant *S. typhimurium* (Figure 1), which laid the foundation for the following experiments. After modeling, some of the mice developed loose hair, poor spirits, and soft stools. From the beginning to the end of modeling, we recorded the weight changes of all the mice. At the time of sampling, photographs of the intestines in the experimental animals were made; comparisons between treatment and negative controls were noted. As shown in Figure 2A, the mice in control group were significantly lower in weight. In Figure 2B,C, the intestine of the control group (b) was thinner than in the normal group (a). After Mdc administration (c) and ceftriaxone sodium treatment (d), intestinal tissue of infected animals appeared to improve significantly.

### *Musca domestica* Cecropin (Mdc) Decreases Bacterial Translocation

**FIGURE 3:**
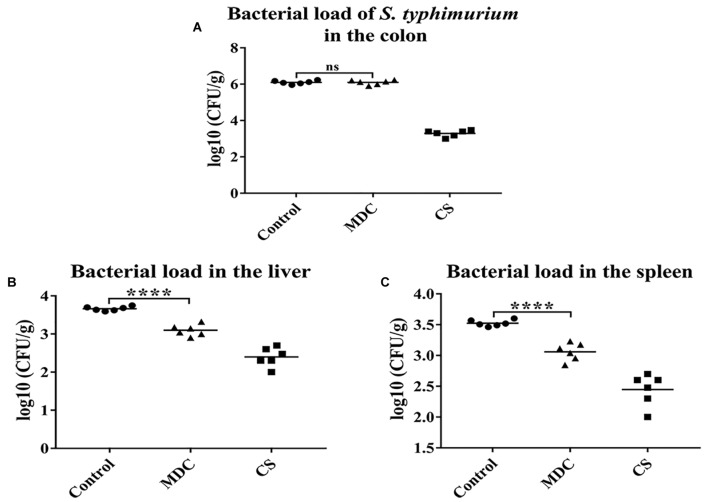
The bacterial load of *Salmonella typhimurium* in the colon and the bactrial load in the liver and spleen were analyzed. Mdc couldn’t decrease bacterial load of *S. typhimurium* in the colon **(A)**. But Mdc significantly ameliorated the bacterial translocation after *S. typhimurium* infection in the liver **(B)** and spleen **(C)**. Values are means ± SEMs, *n* = 6. ns, no significant difference, ^∗∗∗∗^*p* ≤ 0.0001.

Figure 3A showed that there was no significant difference in the number of *S. typhimurium* in feces between MDC group and Control group, indicating that intraperitoneal injection of Mdc did not affect bacterial load of *S. typhimurium* in colon contents. However, in Figure 3B,C, Mdc significantly reduced the number of bacterial translocation in liver and spleen.

### Mdc Reduces Colonic Mucosal Barrier Damage

**FIGURE 4:**
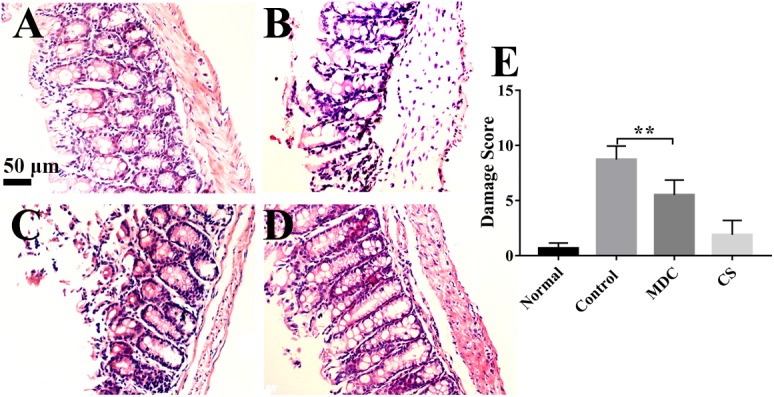
Representative micrographs of HE staining show the colon of normal group **(A)**, control group **(B)**, Mdc group **(C)**, and Cs group **(D)**. Histological damage was scored using the criteria of Appleyard and Wallace. The damage score **(E)** intuitively indicates that Mdc could reduce colon tissue damage. Values are means ± SEMs, *n* = 6, ^∗∗^*p* ≤ 0.01.

As Figure 4 shows, microscopically, the colonic epithelial cells of normal group (Figure 4A) had a normal appearance, the glands of lamina propria were compact and regular, the mucosa and submucosa were clear and goblet cells were abundant; no inflammatory cell infiltration or ulcer formation was observed. In control (Figure 4B) and Mdc groups (Figure 4C), the epithelial cells of colonic mucosa were exfoliated, the glands were arranged in a disorderly fashion, inflammatory cells infiltrated the mucosa and submucosa, and erosion, destruction of glands, structural disorder, damage of epithelial integrity, loss of goblet cells were observed. The pathological signs in colon mucosa of Mdc group animals were less pronounced than those in the infection (positive) control group; however, intestinal tissues in the former group did show visible signs of infection (Figure 4D). The damage score (Figure 4E) intuitively summarizes the contents of the above four photos. These results show that while Mdc treatment can mitigate the severity of *S. typhimurium*-induced mucosa damage, its application does not completely reverse *S. typhimurium*-caused physiopathology.

**FIGURE 5:**
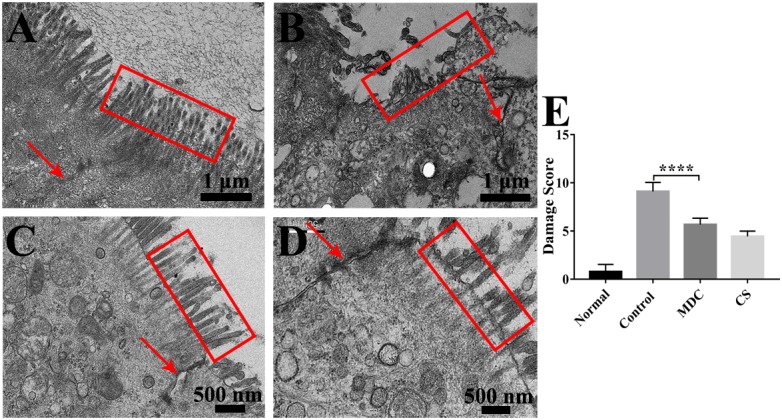
The ultrastructure of the colonic epithelium of mice in the normal group **(A)**, control group **(B)**, Mdc group **(C)**, and Cs group **(D)** under the transmission electron microscope. The red arrows indicate tight junctions (TJs) and the colonic microvilli are surrounded by red outline. The damage score **(E)** from ultrastructure of colon tissues shows the positive effect of Mdc on colon tissue damage. Values are means ± SEMs, *n* = 6, ^∗∗∗∗^*p* ≤ 0.0001.

In order to further observe the effects of Mdc on colonic mucosa damage caused by *S. typhimurium*, TEM was performed. It could be seen very intuitively from Figure 5 that the microvilli in the colonic epithelium of the normal group (Figure 5A) were well-distributed, and the size of cells was basically the same and the arrangement was compact, forming a complete tight junction. The control group (Figure 5B) showed widespread damage to microvilli as well as tight junction structure disruption, and epithelial cell death. Microphotographs corresponding to the Mdc group and Cs group were shown in Figure 5C,D, in which clearly show that Mdc application reduces the level of *S. typhimurium* induced colonic epithelial damage. Furthermore, the damage score (Figure 5E) from ultrastructure of colon tissues also shows the positive effect of Mdc on colon tissue damage.

### Inhibition of Inflammatory Response

**FIGURE 6:**
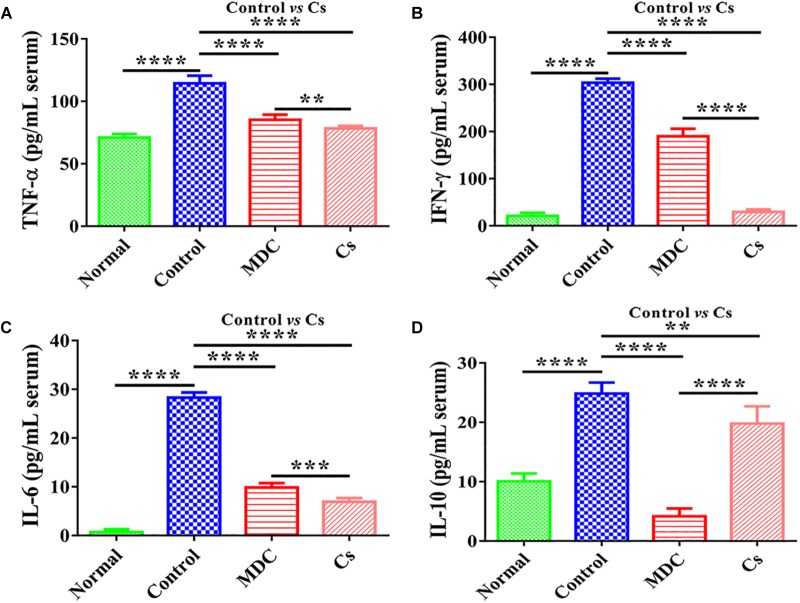
The concentrations of TNF-α **(A)**, IFN-γ **(B)**, IL-6 **(C)**, and IL-10 **(D)** in the serum of 4 groups’ different mice. Values are means ± SEMs, *n* = 6. ^∗∗^*p* ≤ 0.01, ^∗∗∗^*p* ≤ 0.001, ^∗∗∗∗^*p* ≤ 0.0001.

The contents of inflammatory factors in the serum of mice were conducted to evaluate the anti-inflammatory activity of Mdc. The results of TNF-α, IFN-γ, IL-6, and IL-10 concentrations in mice serum are shown in Figure 6. TNF-α, IFN-γ, IL-6, and IL-10 concentrations in serum were significantly decreased by exposure to Mdc than in the control group.

### Suppression of Oxidative Stress in Colon

**FIGURE 7:**
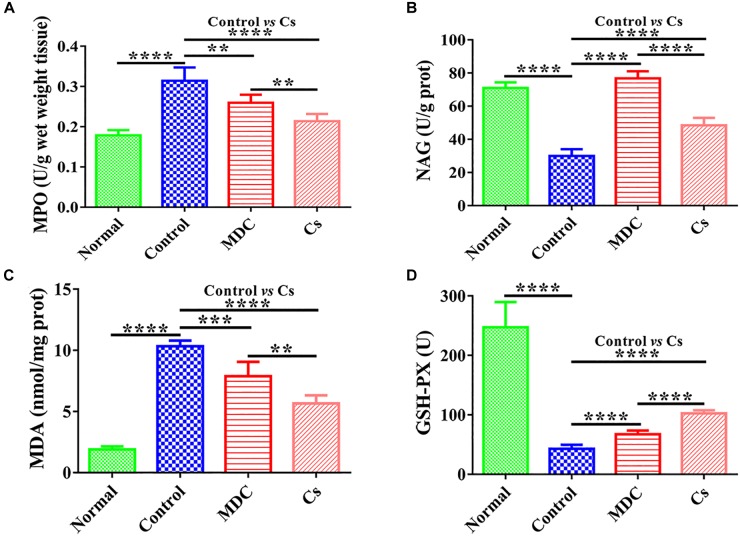
The activities of MPO **(A)**, NAG **(B)**, GSH-Px **(D)**, and the level of MDA **(C)** in the colon of 4 groups’ different mice. Values are means ± SEMs, *n* = 6. ^∗∗^*p* ≤ 0.01, ^∗∗∗^*p* ≤ 0.001, ^∗∗∗∗^*p* ≤ 0.0001.

Measuring intestinal tissue indicators was the basis for determining intestinal epithelial health, and colon was the most suitable with regard to our model. MPO, NAG, MDA, and GSH-Px in colon were measured. Figure 7 shows that Mdc could greatly promote GSH-Px and NAG activity as compared with the control group. Furthermore, Mdc largely depressed MPO activity and MDA levels.

### Three Different Methods Jointly Verify Increasing Tight Junctions and Reducing Inflammation in Colon Induced by Mdc

**FIGURE 8:**
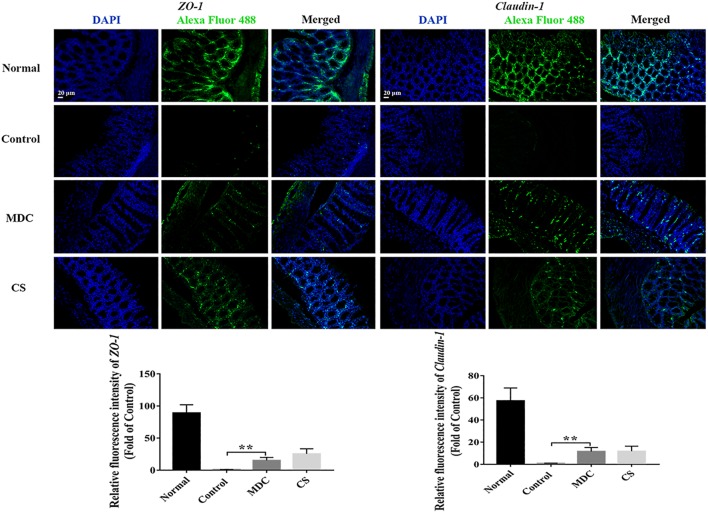
Immunofluorescence analysis of ZO-1 and Claudin-1 in colon of 4 groups. Values are means ± SEMs, *n* = 3, ^∗∗^*p* ≤ 0.01.

**FIGURE 9:**
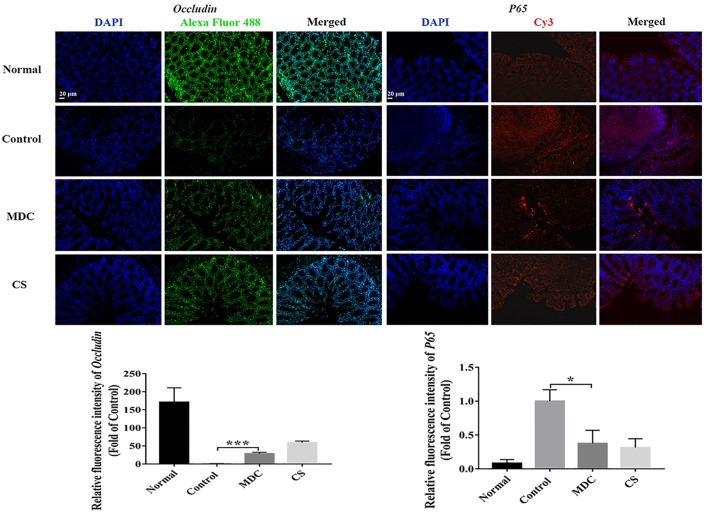
Immunofluorescence analysis of Occludin and P65 in colon of 4 groups. Values are means ± SEMs, *n* = 3. ^∗^*p* ≤ 0.05, ^∗∗∗^*p* ≤ 0.001.

**FIGURE 10:**
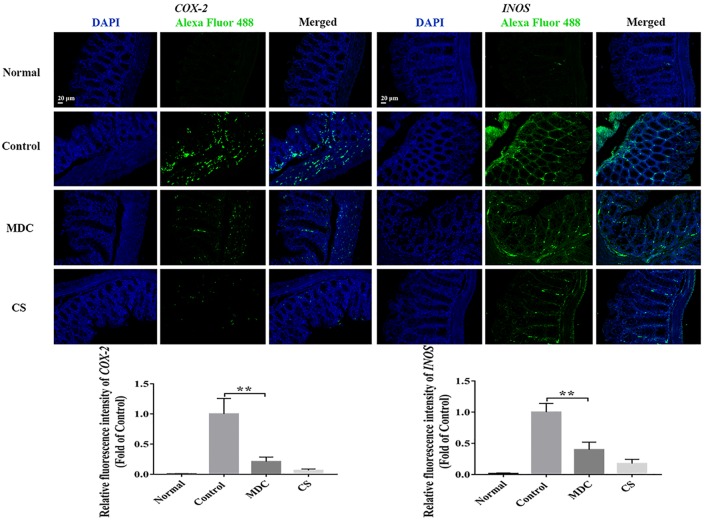
Immunofluorescence analysis of COX-2 and INOS in colon of 4 groups. Values are means ± SEMs, *n* = 3, ^∗∗^*p* ≤ 0.01.

The expression of ZO-1, Claudin-1, Occludin, P65, COX-2, and INOS proteins in colon were examined using immunofluorescence analysis. It could been seen clearly from Figure 8–10 that while comparing with the control group, the ZO-1, Claudin-1 and Occludin proteins in Mdc group showed increased significantly. On the contrary, the P65, COX-2 and INOS proteins expressed less in Mdc group. It was shown that the normal group had the highest expressions of the ZO-1, Claudin-1 and Occludin. The normal group also showed the lowest expression levels of P65, COX-2, and INOS proteins. There were notable increases in the expression levels of the ZO-1, Claudin-1 and Occludin proteins with significant decreases of P65, COX-2, and INOS proteins in the Mdc treated group as compared to the control group.

**FIGURE 11:**
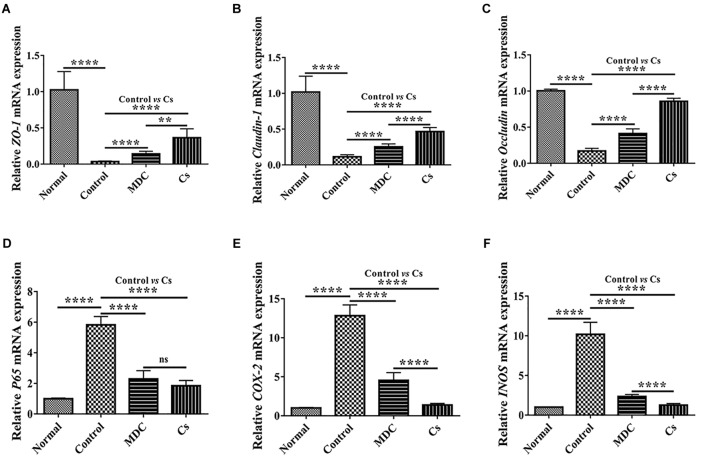
Real-time quantitative PCR detection of ZO-1 mRNA **(A)**, Claudin-1 mRNA **(B)**, Occludin mRNA **(C)**, P65 mRNA **(D)**, COX-2 mRNA **(E)**, and INOS mRNA **(F)** in colon of 4 groups. Values are means ± SEMs, *n* = 6. ^∗∗^*p* ≤ 0.01, ^∗∗∗∗^*p* ≤ 0.0001, ns, no significant difference.

mRNA expression levels of ZO-1, Claudin-1, Occludin, P65, COX-2, and INOS in colon were examined through Real-time quantitative PCR. As shown in the Figure 11, the normal group possessed the highest expression level of ZO-1 (Figure 11A), Claudin-1 (Figure 11B), and Occludin (Figure 11C) as well as the lowest expression levels of P65 (Figure 11D), COX-2 (Figure 11E), and INOS (Figure 11F), followed by the Cs group, the Mdc group and the control group. The results of Real-time quantitative PCR detection were consistent with the results of immunofluorescence analysis.

**FIGURE 12:**
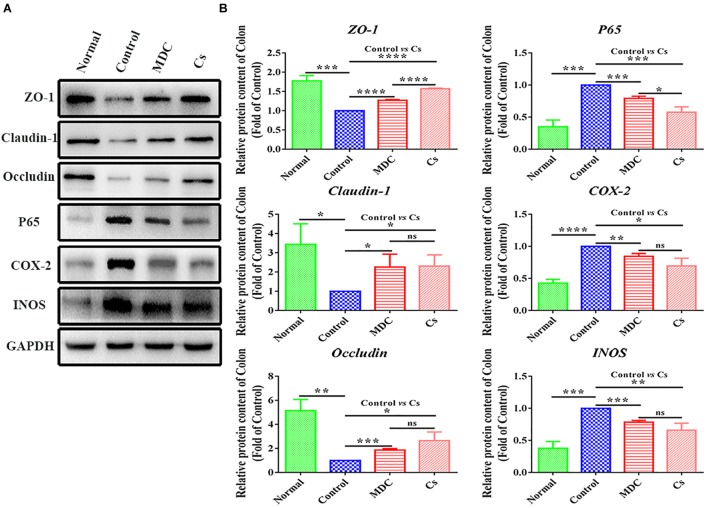
Western blot analysis of ZO-1,Claudin-1, Occludin, P65, COX-2, INOS protein expression in relative to GAPDH in the colon of 4 groups **(A,B)**. Values are means ± SEMs, n = 3. ^∗^*p* ≤ 0.05, ^∗∗^*p* ≤ 0.01,^∗∗∗^*p* ≤ 0.001, ^∗∗∗∗^*p* ≤ 0.0001, ns = no significant difference.

In order to further verify the results of immunofluorescence analysis and Real-time quantitative PCR detection, we carried out western blot analysis. The results were shown in Figure 12 represented protein band and protein expression in relative to GAPDH (fold of control group), respectively. The results of western blot analysis showed the changes in the expression levels of ZO-1, Claudin-1, Occludin, P65, COX-2, and INOS similar to the results of immunofluorescence analysis.

### Mdc Ameliorates Intestinal Flora Imbalance Closely Related to Tight Junctions

**FIGURE 13:**
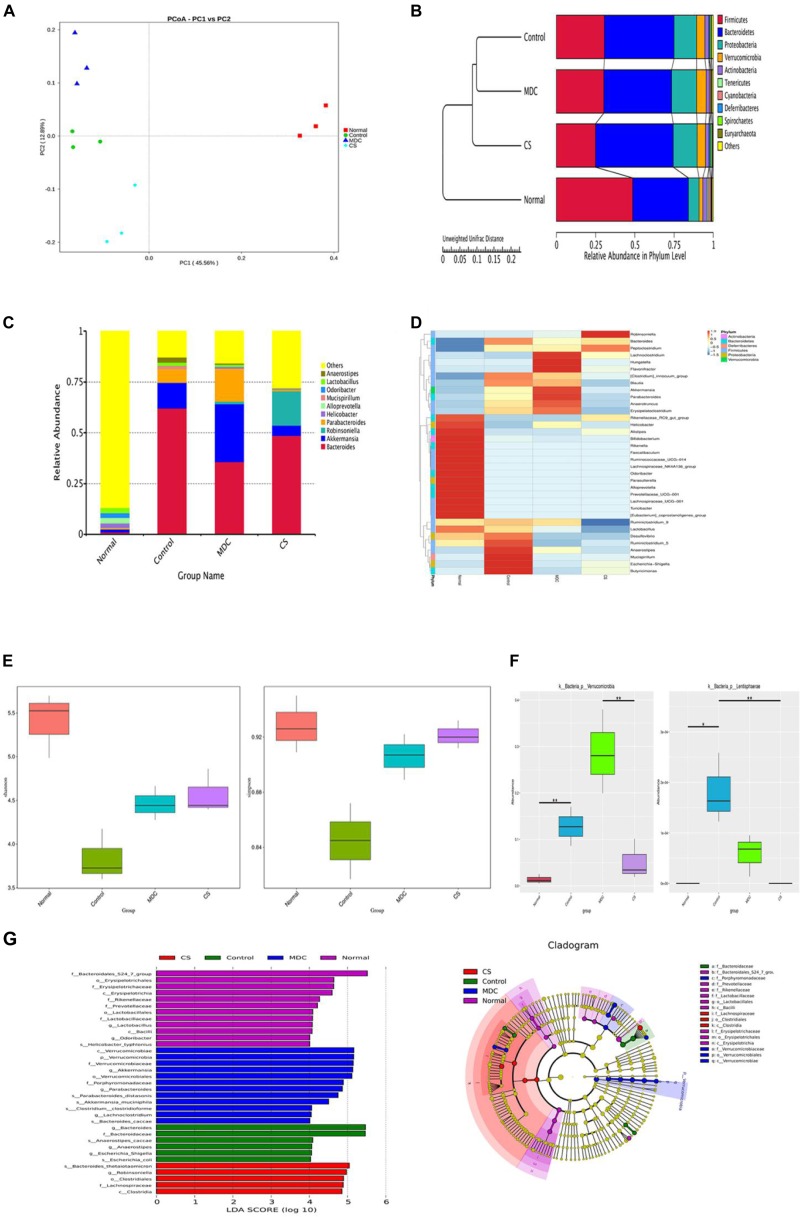
Microbiota composition in the feces were analyzed using 16s rDNA sequencing (*n* = 3 for each group). Unweighted unifrac-based PCoA analysis, each point in the graph represents a sample and the same group of samples is represented in the same color **(A)**. Unweighted unifrac-based UPGMA clustering tree **(B)**. Relative abundance of each group at the genus level **(C)**. Species richness on the level of genus, the horizontal axis represents the sample information, and the vertical axis represents species annotation information, the left side of the cluster tree represents the species cluster tree, the corresponding value of the intermediate thermal map is the *Z* value obtained after normalization of the relative abundance of each row species **(D)**. Analysis of Alpha diversity index (shannon and simpson) among groups **(E)**. MetaStat analysis to find species with significant difference among groups in the phylum level **(F)**. ^∗^*q* ≤ 0.05, ^∗∗^*q* ≤ 0.01. Linear discriminant analysis (LDA) plots highlighting significantly different characteristic taxons among 4 groups’ microbiota, only taxa with a value of LDA score of more than 4 are shown (**G**, Left). Taxonomic cladogram of 4 groups’ bacterial fecal samples (**G**, Right).

In order to test whether Mdc regulated gut microbiota and characterize the modifications in the intestinal microbiota composition, we performed 16S rDNA sequencing in fecal samples of mice. The regulatory role of Mdc on the intestinal microecological system was indicated by the α, β diversity and OTUs analysis of the fecal samples. Unweighted unifrac-based PCoA analysis revealed a distinct clustering of microbiota composition for each group (Figure 13A). Closer lower sample distance implied a similar species composition; the normal group was relatively distant from the other three group cluster. From the UPGMA clustering tree (Figure 13B), the community structure of the Mdc group was more similar to the Cs group and the normal group than the control group, thus indicating that Mdc exerted considerable effects on the intestinal flora. The 10 most abundant bacterium in the level of genus were compared among groups (Figure 13C). The composition of intestinal bacterial species of Mdc group was dramatically changed compared with the other groups. Abundance of *Bacteroides* was decreased in the Mdc group with same trend as the normal group and Cs group. Interestingly, unlike the Cs group and normal group, Mdc treatment increased the abundance of *Akkermansia* and *Parabacteroides*. Heatmap and cluster analysis of the 35 most abundant genera were conducted based on the abundance of species annotation information (Figure 13D). Shannon and Simpson (Figure 13E), important components of alpha diversity index, are used to estimate the diversity of microbial communities in the samples. Compared with Shannon and Simpson indices of four groups, the two indices in the normal group were the biggest, and the second was the Cs group near the tertiary Mdc group. These results indicated that the Mdc could significantly increase species diversity compared with the control group. In order to study the species with significant difference among groups, the *p* value was obtained by hypothesis testing based on the phylum level species abundance table, and the *q* value was obtained by correcting the *p* value. Finally, the species with significant difference were selected according to the *q* value. As shown in Figure 13F, *Verrucomicrobia* and *Lentisphaerae* in the phylum level displayed significant differences among 4 groups. LEfSe (LDA Effect Size) analysis (Figure 13G, Left) demonstrated that Mdc increased the relative proportions of bacteria from the class *Verrucomicrobiae*, phylum *Verrucomicrobia*, family *Verrucomicrobiaceae*, genus *Akkermansia*, order *Verrucomicrobiales*, while the bacteria from the genus *Bacteroides* and family *Bacteroidaceae* had the greatest growth in the control group. The cladogram (Figure 13G, Right) showed that organisms in phylum *Verrucomicrobia* were mainly enriched in the Mdc group. For the normal group and Cs group, the cladogram also corroborated the LEfSe analysis results.

**FIGURE 14:**
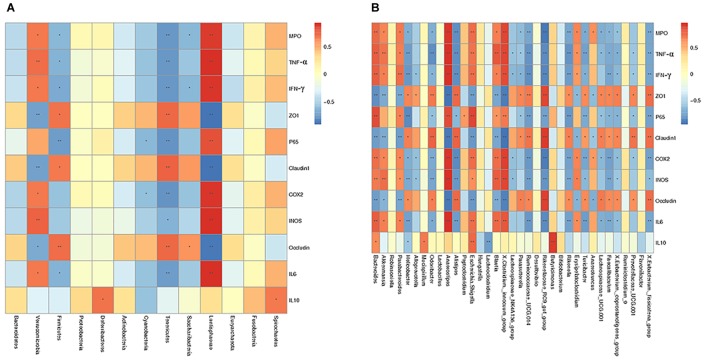
Spearman correlation analysis was conducted to determine the relationship between various factors and species at the phylum level **(A)** as well as genus level **(B)**. The corresponding value of the intermediate thermogram is Spearman correlation coefficient r, between -1 and 1, *r* < 0 is negative correlation, *r* > 0 is positive correlation. ^∗^*p* ≤ 0.05, ^∗∗^*p* ≤ 0.01.

To further investigate how intestinal flora affected colonic mucosal injury caused by *S. typhimurium*, correlation analysis including Spearman sequential correlation analysis and Mantel test were conducted. IL-6, INOS, COX-2, P65, TNF-α, IFN-γ and MPO were positively correlated with phylum *Verrucomicrobia* and *Lentisphaerae* enriched in the control group, with which ZO-1, while Claudin-1 and Occludin were negatively correlated. There was a positive correlation between ZO-1, Claudin-1, Occludin and phylum *Tenericutes* and *Firmicutes*, and IL-6, INOS, COX-2, P65, TNF-α, IFN-γ, and MPO were negatively correlated with these clades. At the genus level, it should be noted that genus *Akkermansia* and genus *Bacteroides*, *parabacteroides* had a positive correlation with IL-6, INOS, COX-2, P65, TNF-α, IFN-γ and MPO (Figure 14).

Mantel test was used to calculate correlation between combination factors and microbial community data. As shown in Table 2, Mantel Test based on relative abundance in the level of genus and OTUs illustrated that the combination factors of IL-10 + ZO-1 + Occludin + Claudin-1 and ZO-1 + Occludin + Claudin-1 played the greatest role in the genus relative abundance and microbial species composition.

**Table 2:** Mantel test based on relative abundance in the level of genus (above) and OTUs (below).

Variable	*r*	*p*
MPO + P65 + COX-2 + INOS	0.1682	0.155
	0.4187	0.009
TNF-α + IFN-γ + IL-6 + IL-10	0.1929	0.094
	0.3846	0.018
ZO-1 + Occludin + Claudin-1	0.7420	0.001
	0.9078	0.001
MPO + P65 + COX-2 + INOS + ZO-1 + Occludin + Claudin-1	0.4199	0.004
	0.6729	0.002
MPO + P65 + COX-2 + INOS + TNF-α + IFN-γ + IL-6 + IL-10	0.2120	0.090
	0.4349	0.007
MPO + P65 + COX-2 + INOS + TNF-α + IFN-γ + IL-6	0.2083	0.107
	0.4580	0.012
IL-10 + ZO-1 + Occludin + Claudin-1	0.7520	0.001
	0.7883	0.001
MPO + TNF-α + IFN-γ + ZO-1 + P65 + Claudin-1 + COX-2 + INOS + Occludin + IL-6 + IL-10	0.2765	0.052
	0.5129	0.011


*Variable represents the combination factors information, *r* is the correlation coefficient, and *p* is the significance test of *P* value. The greater the *r* value indicates the greater correlation between combination factors and species richness information, *P* < 0.05 shows significant statistical significance.*

## Discussion

There is an impressive amount of literature on how AMPs maintain intestinal homeostasis such as stimulation of mucus synthesis, promoting the production of tight junction proteins and immune modulation (Brown et al., 2012; Ma et al., 2012; Tang et al., 2013; Kiatsurayanon et al., 2014). However, only very limited even no information is available on how AMPs affect intestinal microflora to maintain intestinal mucosal barrier with normal function (Pound et al., 2015; Fusco et al., 2017). Here, we elucidate how AMP Mdc improve the damage of colonic mucosal barrier by regulating intestinal flora for the first time, meanwhile other common functions of AMPs have also been verified. The present study provided convincing evidence that Mdc could markedly alleviate intestinal mucosal injury by not only depressing inflammatory response and oxidative stress response but also more importantly ameliorating zonula occludens and bacterial flora distribution.

*Salmonella enterica* is one of the leading causes of zoonotic foodborne disease worldwide. *S. typhimurium* had always been the top three pathogens isolated from foodborne diseases and food poisoning cases in China, among which *S. typhimurium* has the highest detection rate (Yang et al., 2013). It is an invasive bacterium that mainly invades small intestine and colon (Winter et al., 2010). Intestinal mucosal barrier injury frequently occurs following *S. typhimurium* challenge (Pohl et al., 2018). Although there are many Salmonella infection models that can be used to study different types of intestinal injury (Santos et al., 2001), the *S. typhimurium* infection model used in this experiment is usually used to establish colonic mucosal lesions (Manja et al., 2003). Therefore, the colonic mucosal damage caused by *S. typhimurium* infection was discussed in this study. In addition, according to Xia’s et al. (2015) research and preliminary experimental results, AMP Mdc may also improve ileal injury induced by *S. typhimurium*, which will be performed further in the future experiments. This model was very stable that symptoms of *S. typhimurium* infection were found in mice of model group and there was no death in mice. In the present study, H&E staining and intestinal TEM showed that Mdc significantly improved intestinal mucosal barrier damage induced by *S. typhimurium*. Furthermore, the decrease of bacterial translocation also fully demonstrated this role of Mdc.

There are many connections between intestinal mucosal epithelial cells, in which TJs are the most important, and the most important components of TJs are tight junction proteins such as ZO-1, Claudin-1 and Occludin (Betanzos et al., 2013). These proteins play pivotal roles in maintaining the integrity of tight junction between cells and the normal function of epithelial cell barriers. Abnormal expression of these protein cause barrier dysfunction, leading to dysfunction of tight junction and increased tissue permeability, which is one of the characteristics of inflammatory bowel disease (Walsh-Reitz et al., 2005; Förster, 2008; Mir et al., 2016; Li et al., 2018; Lucke et al., 2018). In the present study, the results of immunofluorescence analysis, Real-time quantitative PCR detection and western blot analysis demonstrated that Mdc could improve diseased intestinal tight junction by up-regulating the level of tight junction proteins ZO-1, Occludin and Claudin-1.

The intestinal mucosa has a variety of mechanisms for maintaining homeostasis, preventing excessive inflammatory responses (Wang and Colgan, 2017). Intestinal mucosal barrier injury could stimulate the occurrence of proinflammatory events (Turner, 2009; Capaldo et al., 2017). The intestinal epithelium is central to coordinating both inflammation and resolution (Ivanov et al., 2010). A recent study reported that the regulation of epithelial barrier function could inhibit intestinal inflammation (Shi et al., 2018). In addition, inflammation is also a key factor affecting intestinal mucosal barrier (Kamekura et al., 2015). Therefore, the decreases of TNF-α, IFN-γ and IL-6 induced by Mdc in our study could not only reflect its anti-inflammatory activity but also serve to protect the mucosal barrier. IL-10 is a multicellular and multifunctional cytokine and recognized as an inflammatory and immunosuppressive factor, which regulates cell growth and differentiation, participates in inflammatory and immune responses (Huang et al., 2018). Normal, Mdc and Cs group did not increase IL-10, which may be related to the intensive immune status of mice. In addition, IL-17 is an important component of the inflammatory response to infection with *Salmonella serotypes*. There will be a marked upregulation of IL-17 expression with the *Salmonella* infection (Manuela et al., 2007). IL-1β also greatly contributes to innate immune defenses against *S. typhimurium* infection (Bärbel et al., 2006). These important *Salmonella* infectious related cytokines will be further studied in the follow-up of this research.

Antioxidant activity is one of the likely mechanisms involved in amelioration of gut barrier dysfunction (Assimakopoulos et al., 2004; Chaudhry et al., 2016). Myeloperoxidase (MPO) is a constituent enzyme found principally in neutrophils that results in the formation of hypochlorous acid. Its presence in neutrophils allows it to be extracted from tissues; levels of this acid is directly proportional to neutrophil numbers (Liu et al., 2010). Maleic dialdehyde (MDA), a reliable marker of lipid peroxidation, has a positive correlation with levels of oxidative stress. Glutathione peroxidase (GSH-Px) is an important peroxide decomposing enzyme widely found in the body. After Mdc administration, MPO activity and MDA levels were significantly decreased, while the GSH-Px activity had a corresponding increase, indicating that Mdc had the ability to increase the anti-oxidative capacity to prevent intestinal barrier dysfunction, if only to a limited degree.

It has been reported that intestinal permeability and colitis severity are altered by the intestinal microbiota in mice (Llewellyn et al., 2018). Furthermore, previous studies suggested that the composition of the gut microbiota shapes the colon mucosal barrier (Jakobsson et al., 2015). 16S rDNA sequencing indicated that Mdc possessed considerable regulatory effects on the intestinal flora; its application brought the flora of diseased intestines to profiles more similar to that in normal group. Mdc treatment improved the abundances of *Akkermansia* and *Parabacteroides* and species composition diversity and decreased the abundance of *Bacteroides*, indicating reduction of intestinal inflammation and injury (Ramanan et al., 2016; Neyrinck et al., 2017). The increased phylum *Verrucomicrobia* and decreased phylum *Lentisphaerae* induced by Mdc showed significant differences compared with the control group. Although increase of phylum *Verrucomicrobia* was not in line with the expected trend to normal, we suspected that this may be one of the manifestations of Mdc’s unique role. Spearman sequential correlation analysis showed the apparent opposite between tight junction and inflammation. Moreover, the Mantel test also proved that tight junction proteins were closely related to intestinal flora. So the intestinal flora regulation of Mdc was mainly achieved by regulating tight junction. Tight junction dysfunction has been linked to a variety of local and systemic diseases (Buckley and Turner, 2018), which may have a decisive effect on intestinal flora disorder, inflammatory response and oxidative stress induced by *S. typhimurium*.

Increased epithelial permeability is observed in inflammatory states, and Matthias Bruewer clarified that IFN-γ produces a leaky epithelial barrier by inducing macropinoytosis of TJ proteins (Bruewer et al., 2005). In DSS colitis, the loss of tight junction proteins and increased permeability preceded the development of significant intestinal inflammation suggesting that alterations in the TJ complex occur before the intestinal inflammation and not as a consequence of it (Poritz et al., 2007). Oxidative stress is also one of the ways to alter tight junction proteins, thereby enhancing intestinal dysfunction (Ha et al., 2016). The reduction of oxidative stress may upregulate tight junction protein expression (Wu et al., 2018). Intestinal inflammation and oxidative stress may be partly caused by intestinal bacteria, but they also affect the environment, and changes bacterial population profiles (Ren et al., 2018). In our study, the results also showed that both inflammatory factors and tight junction proteins are closely related to intestinal flora. Altogether, there is a complex interrelationship among them (Tlaskalová-Hogenová et al., 2011).

In conclusion, these data suggested that Mdc had an obvious protective effect on *S. typhimurium* induced colonic mucosal barrier injury in mice. The mechanism may be through inhibiting colonic inflammation and oxidative stress, up-regulating the expression of tight junction proteins ZO-1, Occludin, Claudin-1 in colonic epithelial cells, improving intestinal flora imbalance, reducing the damage of colonic epithelium and intestinal permeability, protecting the tight junction structure of colon mucosa and ameliorating the function of colon mucosal barrier. These findings could enhance our understanding on the effect and mechanism of Mdc on colonic mucosal barrier damage caused by *S. typhimurium* infection and contribute to developing effective therapies in the future.

## Author Contributions

LZ and XL performed the research and wrote the manuscript. SG and ZL collected and analyzed the data. FC, XJ, and JZ participated in the design of the study. AL, ZC, YT, MX, and WL prepared the tissue sections. All authors approved the final version of the manuscript.

## Conflict of Interest Statement

The authors declare that the research was conducted in the absence of any commercial or financial relationships that could be construed as a potential conflict of interest.
